# Cortisol in hair measured in young adults - a biomarker of major life stressors?

**DOI:** 10.1186/1472-6890-11-12

**Published:** 2011-10-25

**Authors:** Jerker Karlén, Johnny Ludvigsson, Anneli Frostell, Elvar Theodorsson, Tomas Faresjö

**Affiliations:** 1 Department of Medical and Health Sciences, Division of Community Medicine. Linköping University, SE-581 83 Linköping, Sweden; 2 Department of Clinical and Experimental Medicine, Division of Pediatrics. Linköping University, SE-581 83 Linköping, Sweden; 3 Department of behavioural science and learning, Division of cognition, development and disability. Linköping University, SE-581 83 Linköping, Sweden; 4 Department of Clinical and Experimental Medicine, Division of Clinical Chemistry. Linköping University, SE-581 83 Linköping, Sweden

**Keywords:** Biomarker, Coping, Cortisol, Hair, Serious life events, Stress

## Abstract

**Background:**

Stress as a cause of illness has been firmly established. In public health and stress research a retrospective biomarker of extended stress would be an indispensible aid. The objective of this pilot study was to investigate whether concentrations of cortisol in hair correlate with perceived stress, experiences of serious life events, and perceived health in young adults.

**Methods:**

Hair samples were cut from the posterior vertex area of (n = 99) university students who also answered a questionnaire covering experiences of serious life events, perceived Stress Scale and perceived health during the last three months. Cortisol was measured using a competitive radioimmunoassay in methanol extracts of hair samples frozen in liquid nitrogen and mechanically pulverised.

**Results:**

Mean cortisol levels were significantly related to serious life events (p = 0.045), weakly negatively correlated to perceived stress (p = 0.025, r = -0.061) but nor affected by sex, coloured/permed hair, intake of pharmaceuticals or self-reported health. In a multiple regression model, only the indicator of serious life events had an independent (p = 0.041) explanation of increased levels of cortisol in hair. Out of four outliers with extremely high cortisol levels two could be contacted, both reported serious psychological problems.

**Conclusions:**

These findings suggest that measurement of cortisol in hair could serve as a retrospective biomarker of increased cortisol production reflecting exposure to major life stressors and possibly extended psychological illness with important implications for research, clinical practice and public health. Experience of serious life events seems to be more important in raising cortisol levels in hair than perceived stress.

## Background

The evidence for stress as a cause for a range of diseases in modern industrial societies has been growing stronger in recent decades. Psychosocial factors like anxiety, social isolation, stressful life events and lack of control over work accumulate during life and increase the risk of premature death or poor mental health [1]. These so called psychosocial stressors ultimately affect the health. The evidence seems to be strongest for depression [2,3] but is also strong for cardiovascular diseases [4,5] and even HIV [6]. More evidence is also emerging regarding other diseases including e.g. infections and autoimmune diseases [7,8].

The role of the social environment for health is evident in public health science [9,10] and for centuries it has been apparent in medicine to include social determinants to understand disease occurrence, health outcomes and prevention [11]. However, how the social environment affects health often appears to be an unspecified black box which remains after investigators have controlled for a range of individual characteristics [9,12]. In this perspective it is important to investigate "upstream" influences on more proximal determinants of health [13]. Stress is suggested to be one of these upstream influences by which health is affected [14].

The physiological mechanisms that mediates stress and their contribution to illness and manifest disease is not fully understood. One plausible and much studied pathway in the stress response is that of the neuroendocrin Hypothalamic-Pituitary-Adrenocortical (HPA) axis, which in turn controls other physiological systems like immune and inflammatory processes. Negative strain, stressors, cause negative affective states, e.g. anxiety, that alter the production of Corticotropin-Releasing Hormone (CRH) in the paraventricular nucleus of the hypothalamus. In response to levels of CRH, Adrenocorticotropic hormone (ACTH) is released from the pitiuitary gland out to the circulating blood ultimately reaching the cortex of the adrenal gland effecting synthesisation of cortisol, the primary effector of HPA-axis activation in humans. High cortisol level has potential deleterious effects. For example it cause prolonged wound healing [15] as well as a significant degree of cognitive impairment [16]. This stress-response through the HPA-axis is thougt to be a major physiologial mechanism through which stressors influence disease risk [17,18].

Currently, the best way to biologically measure the stress-response is to assess the activity of the HPA-axis through levels of cortisol. Measuring cortisol concentrations in saliva is the most extensively used method of assessing cortisol in stress research as it has some advantages: it is non-invasive and thus less stressful, cost effective, and saliva can be collected by non-medical personnel in many different environments. Unfortunately, current methods of measuring cortisol, including concentrations in saliva, blood or urine only cover a spot time interval and cannot detect stress longitudinally or retrospectively. This drawback makes the assessment of cortisol levels largely dependent on the time and situation of sample collection: Firstly, cortisol levels vary with a natural pulsatile variation during the day (circadian rhythm), which makes the time of the day important [19,20]. Secondly, the levels may also be influenced by situational factors like psychological [21] or physical [22] stressors since they have the ability to elevate cortisol levels up to one hour after onset before returning to baseline [21].

The shortcomings of the present cortisol measuring methods call for a better procedure in determining extended and long-term exposure to stress. Hair has been used as a substrate for measuring environmental agents, drugs or toxins for a number of years [23] and quite recently the ability to retrospectively measure steroid hormones [24,25] including cortisol levels in hair [23,26] was shown. Initial studies on rhesus monkeys showed that cortisol levels in their hair increased when exposed to the stress of relocation and that the cortisol levels also increased in serum and saliva, respectively [27,28]. Later, a few initial studies on humans indicated that the level of cortisol in hair may reflect the activity of the HPA-axis [29,30]. Furthermore, correlations between specific stressors and cortisol in hair in selected groups have been shown. For example, neonates have higher cortisol levels in hair depending on the period of stay at the neonatal intensive care [31]. For a group of patients with chronic pain, higher hair cortisol levels were found compared to a control group [32]. In a proof-of-concept study cortisol levels increased in scalp-near hair segment of pregnant women that corresponded to the well-known increase of blood cortisol levels in the third trimester of pregnancy [33]. An interesting study showed that hair cortisol levels representing three months prior to an acute myocardial infarction among 56 men were significantly higher than in hair from 56 controls who were hospitalised for other indications. This suggests that long term stress may be a contributing factor to myocardial infarction [34]. Recently, a study showed that long-term unemployed individuals had higher hair cortisol levels compared to controls [35].

Cortisol in hair might serve as a longitudinal biomarker of chronic stress and in public health research that would be of great value. However, it is warranted to validate the measurement of cortisol in hair in comparison to other indicators of extended stress. This present investigation serves as a pilot study to develop the methodology of cortisol in hair analysis and to test if cortisol in hair could be measured even in young healthy adults and to relate the cortisol levels to other indicators of stress and health for this group.

### Study aims

Our aim was to investigate whether concentrations of cortisol measured in hair extracts correlate with perceived stress, serious life events and perceived health in a sample of young adults in the general population.

## Methods

### Study population

Medical and nursing students in their second semester at the Faculty of Health Sciences at Linköping University were invited to voluntarily cut hair samples and to anonymously fill in a questionnaire. N = 99 students gave their informed consent to participate in the study. No exclusion criteria were applied, except for those students with crew cut or shaved hair for which hair sampling was impossible.

### Measures

The questionnaire covered the experience of 1) *serious life events *during the last 3 months. This was assessed with a dichotomous (yes/no) question "During the last three months, have you experienced something that you would describe as a serious life event? (e.g., death of a close relative, serious illness or divorce) with an open ended yes-option to describe which kind of event. 2) *perceived health *last 3 months, assessed by a visual analogue scale coded as 0-10 (ranging from "very poor" to "excellent") and 3) *perceived stress *last 3 months assessed with the Perceived Stress Scale (PSS) which is a 14-item instrument assessed on 5-point Likert-scales (ranging from "never" to "very often") ) [36]. Background factors were assessed including sex, age, coloured/permed hair and regular use of prescribed pharmaceuticals last 3 months.

### Extraction of cortisol from hair samples

The hair strands were cut closely to the scalp from the posterior vertex area of the head, which has been shown to have the lowest coefficient of variation [29]. The 3 centimetres of hair closest to the hair roots were analysed, reflecting exposure during the last 3 months since hair grows approximately 1 centimetre per month. Cortisol was measured in methanol extracts of hair using an in-house competitive radioimmunoassay as described in detail below.

Hair samples weighing between 5 mg and 17 mg were minced and extracted in methanol in the following manner. The hair samples were weighed on a Sartorius MC 210p microscale, in 2 mL QiaGen RB sample tubes (for use with QIA cube). A 5 mm steel ball was added to each tube and batches of 5 tubes were put in each solid aluminum holder specially made for the purpose. The holders including the samples and steel balls in the sample tubes were frozen in liquid nitrogen for 2 minutes and rapidly minced in Retch TissueLyser II at 30 Hz for 20 seconds, producing very fine hair powder. One mL pure methanol (Chromasolv, Sigma-Aldrich) was added to each tube and each tube fixed in metal holder on an oblique (5 degrees from the horizontal) plate on a horizontal shaker (Edmund Bühler, type B1) at room temperature, making sure that the steel balls continuously moved back and forth in the sample tubes for at least 10 hours. The tubes were then centrifuged and 700 μL of the supernatant moved to another sample tube for lyophilization in SpeedVac Plus SC210A (Savant) using Edwards XDS 5 vacuum pump.

### Measuring cortisol in the extracted hair samples

The samples were dissolved in 150 μL 0.1 mol/L phosphate buffer, pH 7.4 containing 0.02% bovine serum albumin (BSA) and 0.01% triton X-100, and concentrations of cortisol measured as described earlier by Morelius et al. 2004 [37]. The lowest detectable concentration was 1 nmol/L and the within- and between assay coefficients of variation were 6.1% and 9.3%, respectively, at 10 nmol/L.

### Uncertainty when extracting and measuring cortisol in hair samples

Each of the multiple steps in the weighing, extraction and measurement contribute to the overall uncertainty with sample mass as one of the important variables. Ten replicates of hair samples weighing 1, 2, 3, 4 and 5 mg were extracted and measured by the procedure described above and the coefficient of variation calculated (Table 1). Hair samples of 5 mg or more were needed for maintaining a total within-assay coefficient of variation below 8% for hair extraction and measurement of cortisol by the radioimmunoassay. At least 5 mg of hair samples was subsequently used in the analysis. Taking the binding of cortisol as 100%, the antiserum cross-reacts 137% with 5α-dihydroxycortisol, 35,9% with 21-deoxycortisol, 35,9% with prednisolone but less than 1% with endogenous steroids, based on radioimmunoassay data provided by the manufacturer of the cortisol antiserum and of the radioligand.

**Table 1:** Ten replicates weighing 1, 2, 3, 4 and 5 mg of hair from the same subject (50 samples in all) were extracted, measured and total within-assay coefficient of variation calculated

Weight of hair sample (n = 10 for each weight)	Coefficient of variation %
1 mg	46.2
2 mg	16.1
3 mg	12.4
4 mg	13.6
5 mg	7.9

### Analyses

Statistical analyses were conducted using the Statistical Package for the Social Sciences (SPSS) 16.0 software (Chicago, IL, USA). ANOVA was used for univariate tests, followed by a multivariate regression analysis. A p-value of p < 0.05 was considered statistically significant. Four respondents had cortisol concentrations that were considerably higher than the average, these were considered as outliers and consequently omitted from the further statistical analyses. The Research Ethics Committee at Linköping University approved the study.

## Results

Mean cortisol levels for the studied young adults were 19.93 pg/mg (s.d. 33.35) ranging from 1.45 to 212.03 pg/mg. For males, the mean cortisol levels were 17.76 (s.d. 13.34) and for females 20.55 (s.d. 37.66), no sex difference in this respect (p = 0.726). Increased cortisol levels in hair were found among participants reporting that they, during the last three months, had experienced serious life events (p = 0.045) see Table 2. Also participants reporting lower levels in the 14-item Perceived Stress Scale (PSS) had significantly higher levels of cortisol in hair (p = 0.025, r = -0.061). Cortisol levels were neither affected by coloured/permed hair, intake of prescribed pharmaceuticals nor self-reported health although coloured/permed hair showed a non-significant trend (p = 0,089). Furthermore, on a follow up question concerning pharmaceuticals: "If Yes, which kind?" no one reported the use of oral corticosteroids or other hormonal drugs of probable influence to cortisol levels.

**Table 2:** Stress and health factors associated to cortisol levels in hair (pg/mg)

			Univariate associations				**Multivariate associations **‡	
		n	mean	s.d	r*	**p-value **†	Beta	p-value
**Background factors**:								
Sex	Male	24	17.76	13.32	-	0.726		
	Female	71	20.53	37.66	-			
Age (mean: 22.1 s.d: 4.1)		95			-0.073	0.732		
Coloured/permed hair	Yes	40	13.04	16.01	-			
	No	55	24.78	40.89	-	0.089		
Pharmaceuticals	Yes	28	22.85	44.86	-			
	No	67	18.58	27.19	-	0.570		
**Stress and Health factors**:								
Serious life events	Yes	20	32.98	56.90	-			
	No	75	16.32	22.53	-	0.045	0.212	0.041
Perceived stress (PSS)		95			-0.061	0.025	-0.078	0.448
Perceived Health (VAS)		95			0.038	0.251		

* Pearson correlation

† Statistics ANOVA, cortisol levels as dependent variable.

‡ Multivariate regression model: (df = 4) F = 3.43 p = 0.012, cortisol levels as dependent variable, background factors were not included in the regression model.

In a multiple regression model, indicators of perceived stress, perceived health and experience of serious life events were included. Only the indicator of experiences of serious life events the last three months exhibited an independent and significant (p = 0.042) explanation of increased levels of cortisol in hair.

Four respondents had cortisol concentrations that were considerably higher than the average (692.28/750.27/1090.98 and 2027.33 pg/mg), and were considered as outliers. Despite the anonymous data collection, two of these four outliers could be reached by open announcement. Both of them reported serious psychological problems, anorexia nervosa and severe generalized anxiety disorder with panic attacks, respectively.

## Discussion

This study suggests that measurement of cumulated cortisol in hair can be used as a biological marker of exposure to major life stressors in a retrospective fashion, as hair grows approximately 1 centimetre per month [38]. Experience of serious life events seem to be more important in raising cortisol levels in hair compared to perceived stress as measured with PSS, which was weakly negatively correlated (r = -0.061). Two out of four outliers with extremely high cortisol levels reported severe psychological problems during the last three months, which raises the question whether this new biomarker also may reflect severe psychological illness, although resent studies has shown decreased hair cortisol concentrations in patients diagnosed with generalized anxiety disorder [39].

There are some reports during recent years where this novel method to measure cortisol in hair has been applied [29,30,32,34,35]. Although our laboratory methodology is in concordance with these, we have in this report managed to reduce the amount of human hair necessarily for reliable analysis to 5 mg instead of previously reported 25 to 45 mg [33]. This is of great value as we plan further studies in very young children.

The participants in this study were all young adults and university students, and could be considered as a relatively healthy cohort. Nevertheless, the measurement of cortisol concentrations in hair extracts retrospectively detected stress exposure i.e. experiences of serious life events. Cortisol levels thus seems to be related to serious life events even among young healthy adults, and not only in groups of ill adults who have some sort of known stressor compared to a control group. In one way this approach is a harder task than to be able to spot a difference between an exposed experimental group and a control group. The variable Serious Life Event is something that has happened or not, and it is usual to simply ask for such an event with a Yes/No question. However, one should be aware that the way in which a serious life event is perceived is a rather subjective factor. The influence of serious life events on physical and mental health has a long research tradition for over forty years from the early reports by Holmes and Rahe [40]. Their initial publication 1967 that tried to rate life events was criticized on methodological grounds concerning the usefulness of generalized magnitude ratings of life events. Later this quite simple measurement that is used in this report has been widely used in numerous publications [41]. When stressors like negative life events, and also chronic strains and traumas are measured comprehensively, their damaging impacts on health are substantial [42]. In a recent study, hair samples of PTSD patients had higher cortisol levels than those of traumatized controls [43], which support our findings that serious (traumatic) life events raises hair cortisol levels although traumatic load was assessed with a checklist of traumatic event types, not coded as yes or no. In this study there was also a positive correlation between number of different traumatic event types and hair cortisol levels.

We note that a surprisingly high number of students reported serious life events, 20 of 95 subjects. In this age group, possible explanation could be the illness/death of grandparents but also the divorcing parents.

In our report, the perceived stress measurement PSS [36] was weakly negatively correlated to increased cortisol levels but in the multivariate analyses the indicator of serious life events outperformed this indicator. Also, the PSS scale has been validated for a recall period of 4 weeks, but was used to assess 3 months of stress in the current study, which is a limitation. According to our results, the experience of serious life events seems to be the key factor and more important in raising cortisol levels in hair than perceived stress. There is always a risk for recall and reporting bias for retrospective self-reports [44]. Possibly, this is more conceivable for retrospective self-reports of perceived stress than for retrospective reports of actual life events. Nevertheless, a key point is that we do not really know the concordance between retrospective measured subjective and biological stress reactions.

A possible limitation in this study is that there is a risk of confounders like adrenal diseases not known to the participants, even though this is rather unlikely considering the rarity of the diseases. Almost one third of the participants (29%) reported some kind of longstanding medication but the use of pharmaceuticals were not correlated to levels of cortisol in hair in the present study. However, external contamination of the hair through e.g. use of cortisone-containing medications is unlikely but cannot fully be excluded. Coloured or bleached hair did not affect the concentration of cortisol in our analysis, although possible removal of some cortisol by repeated washing should also be considered, shown by Sauvé et al 2007 [29]. One could argue that the likelihood of removal of some cortisol from hair by repeated hair shampooing/washing would be low considering that studies have shown that washing with a solvent in the assay process seems to be an unnecessary process since it removes only small parts of the of the total hormone extracted [23,26]. But there are also studies showing that there is a "wash out" effect [31].

There are a hypothesis that stress- induced cortisol secretion may contribute to central fat and there by be a link between stress and risk for disease [45]. BMI was not asked for in this study, although it was not the aim of this study we can comment that the subjects were healthy young adults studying Health Sciences and no one (judged by eye sight) had class 1 obesity (BMI >30) or above. One student was very underweight; this was also the student who we later found out to have anorexia nervosa. This case also had extremely high levels of cortisol in hair, and was omitted from the analysis, as stated earlier.

Present biological cortisol markers like saliva, urine and blood suffer from the drawback of only covering spot time periods of up to 24 hours. Therefore, there is a need in stress research for biological markers reflecting long term and extended exposure to stress. Cortisol in hair has the potential of becoming a new indicator for stress exposure of major life stressors over periods of months and could even be used in clinical settings. An obvious advantage of analysing cortisol in human hair is the ease of sample collection and transportation as well as being non-invasive. It is also easy to store samples since hair does not decompose like other body fluids or tissues.

In public health research, a reliable and compelling marker of stress that is easily managed, even in larger population studies, would be an incredible asset in addition to psychological and psychometric tests to measure major stressors.

## Conclusions

We conclude that cortisol measured in hair could serve as a retrospective biomarker of increased cortisol production reflecting exposure of major life stressors and possibly extended psychological illness with important implications for research, clinical practice and public health. The promising results in this study calls for a larger sample collection and controlled studies in the general population.

## Competing interests

The authors declare that they have no competing interests.

## Authors' contributions

JK, JL, TF participated in the study design and coordination and completed the data collection. ET developed the method. JK, JL, AF, ET, TF drafted the manuscript. All authors contributed to analysis and interpretation of data, and read and approved the final manuscript.

## Pre-publication history

The pre-publication history for this paper can be accessed here:

http://www.biomedcentral.com/1472-6890/11/12/prepub
